# Age Predicts Functional Outcome in Acute Stroke Patients with rt-PA Treatment

**DOI:** 10.1155/2013/710681

**Published:** 2013-09-19

**Authors:** Jarin Chindaprasirt, Kittisak Sawanyawisuth, Paiboon Chattakul, Panita Limpawattana, Somsak Tiamkao, Patcharin Aountri, Verajit Chotmongkol

**Affiliations:** ^1^Department of Medicine, Faculty of Medicine, Khon Kaen University, 123 Mitraparp Road, Khon Kaen 40002, Thailand; ^2^Improvement of Physical Performance and Quality of Life Research Group, Khon Kaen University, Khon Kaen 40002, Thailand; ^3^Researches and Diagnostic Center for Emerging Infectious Diseases, Faculty of Medicine, Khon Kaen University, Khon Kaen 40002, Thailand; ^4^North-Eastern Stroke Research Group, Khon Kaen University, Khon Kaen 40002, Thailand; ^5^Stroke Unit, Srinagarind Hospital, Faculty of Medicine, Khon Kaen University, Khon Kaen 40002, Thailand

## Abstract

The standard treatment for acute ischemic stroke is thrombolytic therapy. There is limited data on prognostic factors of acute stroke with thrombolytic therapy particularly in Asian population. Acute ischemic stroke patients who were treated with thrombolytic therapy at Srinagarind Hospital between May 2008 and July 2010 were included. Factors associated with Barthel index more than 80 were studied by multiple logistic regression analysis. There were 75 patients included in the study. The mean NIHSS scores before treatment and at 3 months were 9.16 ± 4.82 and 3.83 ± 4.00, respectively, and median Barthel index at 3 months was 86. Only significant predictor for having Barthel index more than 80 points at 3 months was age (adjusted odds ratio 0.929, 95% confidence interval 0.874, 0.988). Four patients developed intracranial hemorrhage after the treatment (5%), and two died (2.6%). In conclusion, age predicts Barthel index in acute stroke patients with rt-PA treatment.

## 1. Introduction

Acute ischemic stroke is the most common neurological disease and the third leading cause of death in Thailand [1, 2]. The sequel is catastrophic for patient and caregivers and causes economic burden for the country. Approximately 200,000 Thai patients were diagnosed with stroke yearly but less than one percent received thrombolytic therapy in time [3]. 

Thrombolysis with the intravenous recombinant tissue-type plasminogen activator (rt-PA) is now the standard of care for AIS patient with onset of stroke less than 4.5 hours [4, 5]. Male gender was shown to be a predictor in acute stroke patients who received rt-PA [6]. There is limited data on prognostic factors particularly in Asian population. Here, we reported the predictor for functional outcomes of acute stroke in the stroke referral system in Northeast Thailand. 

## 2. Stroke Referral System

Srinagarind hospital is a 900-bed university hospital situated in Khon Kaen province, the central part of Northeastern Thailand. It served as a referral center for all provinces in this region. The stroke fast track program was initiated in 2007, and the referral system was complete in January 2008. The stroke network cover 63 primary or community (30 to 90 beds), 4 secondary (120 beds), and 1 tertiary (500 beds) hospitals in 4 provinces including Khon Kaen, Roi Et, Mahasarakam, and Kalasin. Nevertheless, other hospitals in nearby provinces can refer the stroke patients to our hospital if the arrival time is less than 4.5 hours.

## 3. Materials and Methods

We enrolled all consecutive patients diagnosed as acute ischemic stroke or transient ischemic attack who treated at the stroke fast track program, Srinagarind hospital. The study duration was 1 May 2008 to 31 July 2010. Medical records of eligible patients were studied. Baseline characteristics, door-to-needle time (time from the patient's arrival in the emergency room to intravenous rt-PA), onset-to-treatment time (OTT), The National Institute Health Stroke Scale (NIHSS) score, Barthel index (0–100 points), physical signs, laboratory results, and clinical outcomes after therapy and three months were recorded.

Descriptive statistics were used to describe baseline characteristics and the Barthel index, and paired *t*-test was used to test the differences of the NIHSS score before and after treatment. Predictors for having Barthel index at 3 months more than 80 points were executed by multiple logistic regression analysis. Potential clinical factors and factors with univariate *P* value less than 0.20 were included in the final model. Ethics approval was provided by Ethic Committee of Medicine Faculty, Khon Kaen University, under the respect of Helsinki Declaration.

## 4. Results 

There were 626 acute stroke patients presented to our emergency department during study period. Of those, 155 patients (24.76%) were started on the stroke fast track program, and 75 patients (48.39%) were eligible for intravenous thrombolytic therapy. Reasons for not giving thrombolysis are unclear onset, arrival time after stroke onset more than 4.5 hours, no written consent from relatives, and contraindication to rt-PA treatment. Baseline characteristics of all patients are compared with a study from Chulalongkorn hospital and the NINDS study (Table 1). Most clinical variables were comparable to the other two studies except the low rate of Asian patients in the NINDS study.

The majority of patients had normal brain CT scan (53%), and 13% of patients had right cerebral artery infarction from the brain scan. The standard dose of rt-PA (0.9 mg/kg) was delivered to 67 cases, while the remaining 8 cases received lower dose at 0.6 mg/kg due to older age (age more than 80 years). The mean arrival time after stroke onset and door-to-needle time was 109 minutes (ranged 43–175 min) and 42 minutes (ranged 20–67 min), respectively. 

The NIHSS score was dramatically improved after thrombolytic therapy. The mean NIHSS score decreased from 9.16 ± 4.82 prior to treatment to 3.83 ± 4.00 at 3-month followup (*P* value ≤0.001). The median Barthel index, which is used to evaluate patient's function of daily activity, was 86 points at 3-month followup. Only 69 patients had Barthel index values at 3 months. Of those, 41 patients (60.29%) had Barthel index more than 80. Clinical features of patients with Barthel index more than or equal to 80 and less were shown on Table 2. The median age in a group with Barthel index more than 80 was significantly lower than another group. After adjusted by other factors, only significant predictor for having Barthel index more than 80 points at 3 months was age (adjusted odds ratio 0.929, 95% confidence interval 0.874, 0.988), Table 3.

Bleeding was found in 9 patients (12%). Five patients (6.67%) had bleeding at other sites, and four patients (5.33%) developed intracerebral hemorrhage. Two patients (2.66%) died from intracerebral hemorrhage.

## 5. Discussion

Less than 1% of stroke patients in developing countries received the standard thrombolytic therapy. The reasons for not receiving rt-PA include unawareness of the physicians, the transportation problem and the availability of the drug [10]. In this study, 11% of acute stroke patients (75 of 626 patients) enrolled to our stroke fast track. Similarly to other studies [8, 11], rt-PA treatment generally improved both acute and long term outcomes. The NIHSS score was statistically improved after the rt-PA treatment. The mean NIHSS score at baseline in our study was somewhat lower than other studies from Bangkok area (9 versus 20 and 15) [9, 11]. At 3 months after treatment, the mean Barthel index was 86 points. The rates of symptomatic and fatal intracerebral hemorrhage were comparable to other studies both in Thailand and other countries. In this study, the door-to-needle time was 42 minutes, less than those reported in two studies from Bangkok (Table 4) [7]. The OTT time of our study was also shorter, in spite of the fact that patients needed to be transferred from other hospitals or stroke referral system. This might be due to the awareness of the stroke referral system and better commute in this region.

Barthel index is used to evaluate functional status of stroke patients independently in terms of activity daily of life such as bathing or walking. The highest score is 100 indicating good functional status [12]. The rt-PA has been shown to increase number of patients minimally dependent or independent by 12% compared to placebo [8]. In this study, those patients with Barthel index more than 80 had lower median age (Table 2). By the multivariate logistic regression analysis, the only predictor for Barthel index more than 80 points at 3 months was age. Increasing age of one year, the likelihood to have Barthel index more than 80 points at 3 months reduced by 7%. Some studies found that presence of atrial fibrillation, diabetes, glucose level, NIHSS score at admission were predictors of stroke outcomes [13–15]. In a large clinical series, atrial fibrillation is associated with a worse functional prognosis and higher mortality rate [16]. It was found more frequent in Barthel index < 80 group (29.6%) than in Barthel index > 80 group (19.5%). We however did not find these associations in our study.

The limitations in this study include retrospective study design and small number of patients. There were some missing data due to missing data from the patient chart. Even though both limitations exist, the significant predictor of stroke outcome is still found.

## Figures and Tables

**Table 1:** Baseline characteristics of the subjects in the study compared to Chulalongkorn hospital series and NINDS study.

	This study	Chulalongkorn [7]	NINDS study [8]
Age (mean ± SD)	64.60 ± 12.66	65.5 ± 12	68 ± 11
Asian race (%)	100%	100%	0.01%
Male	52%	38%	58%
SBP (mmHg)	140.70 ± 22.84	149 ± 28	155 ± 22
DBP (mmHg)	79.41 ± 16.64	85 ± 14	85 ± 13
Initial plasma glucose (mg/dL)	134 ± 46	120 ± 49	149 ± 71
NIHSS score	9 (1–24)	20 (9–32)	14 (1–37)
Underlying diseases		Not available	Not available
Diabetes mellitus	25 (33%)		
Hypertension	35 (46%)		
Dyslipidemia	15 (20%)		
Atrial fibrillation	16 (21%)		
Smoking	38 (50%)		
Previous stroke	8 (10%)		

Note. NIHSS: National Institute Health Stroke Scale.

**Table 2:** Clinical characters of acute stroke patients with intravenous recombinant tissue-type plasminogen activator (rt-PA) treatment by Barthel index less than or equal to 80 or more at 3 months after treatment.

Variables	Barthel index ≤80 *N* = 27	Barthel index >80 *N* = 41	*P* value
Median age	71 (48–87)	65 (27–78)	0.030
Male gender, *N*	18 (66.67)	22 (53.67)	0.286
Median GCS	15 (11–15)	15 (9–15)	0.540
Diabetes, *N*	12 (44.44)	13 (31.71)	0.286
Hypertension, *N*	16 (59.26)	19 (46.34)	0.297
Dyslipidemia, *N*	5 (18.52)	11 (26.83)	0.429
Atrial fibrillation, *N*	8 (29.63)	8 (19.51)	0.336
Smoking, *N*	18 (66.67)	20 (48.78)	0.146
Median NIHSS	8 (4–24)	9 (1–19)	0.377
Median SBP mmHg	137 (90–193)	137 (90–190)	0.380
Median DBP mmHg	78 (60–109)	80 (50–120)	0.374
Median glucose, mg/dL	90 (15–240)	120 (15–240)	0.195

Note. Data presented as median (range) or number (%); GCS: Glasgow Coma Scale; NIHSS: The National Institutes of Health Stroke Scale; SBP: systolic blood pressure; DBP: diastolic blood pressure.

**Table 3:** Factors associated with having Barthel index more than 80 points at 3 months by univariate and multiple logistic regression analysis.

Factors	Univariate logistic regression	Multiple logistic regression
Age	0.944 (0.898, 0.993)	0.929 (0.874, 0.988)
Male gender	1.727 (0.630, 4.735)	0.502 (0.056, 4.468)
Diabetes	0.580 (0.213, 1.585)	0.745 (0.181, 3.068)
Hypertension	0.593 (0.222, 1.587)	0.585 (0.167, 2.051)
Dyslipidemia	1.613 (0.490, 5.312)	1.476 (0.308, 7.070)
Smoking	0.476 (0.174, 1.304)	0.168 (0.017, 1.649)
NIHSS score	0.943 (0.850, 1.046)	0.906 (0.800, 1.026)

Note. NIHSS: The National Institutes of Health Stroke Scale.

**Table 4:** Comparison of main outcomes among studies.

	NINDS [8]	Chulalongkorn [7]	Thammasat [9]	Present study
IV rt-PA rate (%)	N/A	2	21	11
Mean NIHSS score	14	20	15	9
Mean door-to-needle time (min)	N/A	70	54	42
Mean onset-to-treatment time (min)	N/A	137.7	160	109
Symptomatic intracerebral hemorrhage	6.4	5.9	2	5

Note. N/A: not available; rt-PA: intravenous recombinant tissue-type plasminogen activator (rt-PA); NIHSS: The National Institutes of Health Stroke Scale.
